# Letter to the Editor re: Biglari et al. (2016)

**DOI:** 10.1007/s00402-016-2579-5

**Published:** 2016-09-29

**Authors:** Robert Zura, Gregory Della Rocca, Samir Mehta, Hans Goost, R. Grant Steen

**Affiliations:** 1 Department of Orthopaedic Surgery, Louisiana State University, New Orleans, LA, USA; 2 Department of Orthopaedic Surgery, University of Missouri, Columbia, MO USA; 3 Department of Orthopaedic Surgery, University of Pennsylvania, Philadelphia, PA USA; 4 Department of Orthopedic and Trauma Surgery, University Hospital Bonn, Bonn, Germany; 5 Bioventus LLC, 4721 Emperor Blvd., Suite 100, Durham, NC 27703 USA

We read with interest the recent paper by Biglari et al. [1], which reports a prospective observational study of long bone nonunions treated with low intensity pulsed ultrasound (LIPUS). Of the 61 nonunions included in the study, 20 (32.8 %) were described as showing bone consolidation (“successful treatment”) following application of LIPUS. Nonunions for which LIPUS was deemed as “unsuccessful treatment” had a significantly larger defect gap at the time of LIPUS initiation, and were significantly less stable than those in the successfully treated group; such results are thus reflective of fracture characteristics which are contraindicated for LIPUS therapy [2].

The authors pointedly reference the recent work by Zura et al. [3], stating that “the recent study from Zura et al. presents a highly selected patient collective and might result in misleading conclusions”. We believe this statement to be misleading, and would like to take this opportunity to clarify the Zura et al. findings. In that study, data were analyzed for 767 patients with chronic nonunion (>1 year in duration) treated with LIPUS [3]. All patients were drawn from a prospective patient registry required by the US Food and Drug Administration. The registry was open to all patients and every patient who had complete information was assessed, so this population closely reflects the clinical setting. Indeed, orthopedic registry studies are known to be valuable for monitoring patient outcomes in ‘real-world’ scenarios [4]. In a subgroup analysis of 91 patients who were free of surgery in the 90 days prior to LIPUS treatment, the study by Zura et al. [3] observed an 85.7 % heal rate, in sharp contrast to the 32.8 % heal rate reported by Biglari et al. [1].

Biglari et al. also noted the results of a randomized controlled trial by Schofer et al. comparing LIPUS with sham-device treatment of chronic nonunion patients [5]. Although a non-significant difference in the rate of healing between the study groups is cited, Biglari et al. fail to describe that the study duration was just 16 weeks, since the endpoint of that study was not healing in a traditional sense. The primary outcomes of that study were bone mineral density and gap area at the fracture site, both of which were significantly improved by LIPUS versus sham treatment [5].

We note several additional issues with the study by Biglari et al.: (1) compliance with device usage was raised as an important consideration, but compliance rates were not reported; (2) osteitis, which would likely impair healing substantially, was seen in 23 % (14 of 61) of cases, and was significantly more prevalent in the “unsuccessful treatment” group; (3) chronic nonunion was defined as non-healing for >90 days after surgery, which is inconsistent with most other definitions of nonunion [6]; and (4) bone reduction in Biglari et al. (as shown in Fig. 1) appears to be sub-optimal. Furthermore, patients had an average of 3.02 prior surgeries (18 patients had ≥4 surgeries) prior to LIPUS treatment. If we assume that every patient received surgery at presentation, this means that the patients reported in the study [1] had already failed an average of two additional surgeries, yet 32.8 % of these patients healed with LIPUS treatment and no further surgery. Perhaps the findings of Biglari et al. should be viewed as a testament to the power of LIPUS to heal in the context of sub-optimal surgical treatment.
